# WAVE2 Regulates Epithelial Morphology and Cadherin Isoform Switching through Regulation of Twist and Abl

**DOI:** 10.1371/journal.pone.0064533

**Published:** 2013-05-15

**Authors:** Nicole S. Bryce, Albert B. Reynolds, Anthony J. Koleske, Alissa M. Weaver

**Affiliations:** 1 Department of Cancer Biology, Vanderbilt University Medical Center, Nashville, Tennessee, United States of America; 2 Department of Molecular Biophysics and Biochemistry, Yale University, New Haven, Connecticut, United States of America; The Beatson Institute for Cancer Research, United Kingdom

## Abstract

**Background:**

Epithelial morphogenesis is a dynamic process that involves coordination of signaling and actin cytoskeletal rearrangements.

**Principal Findings:**

We analyzed the contribution of the branched actin regulator WAVE2 in the development of 3-dimensional (3D) epithelial structures. WAVE2-knockdown (WAVE2-KD) cells formed large multi-lobular acini that continued to proliferate at an abnormally late stage compared to control acini. Immunostaining of the cell-cell junctions of WAVE2-KD acini revealed weak and heterogeneous E-cadherin staining despite little change in actin filament localization to the same junctions. Analysis of cadherin expression demonstrated a decrease in E-cadherin and an increase in N-cadherin protein and mRNA abundance in total cell lysates. In addition, WAVE2-KD cells exhibited an increase in the mRNA levels of the epithelial-mesenchymal transition (EMT)-associated transcription factor Twist1. KD of Twist1 expression in WAVE2-KD cells reversed the cadherin switching and completely rescued the aberrant 3D morphological phenotype. Activity of the WAVE2 complex binding partner Abl kinase was also increased in WAVE2-KD cells, as assessed by tyrosine phosphorylation of the Abl substrate CrkL. Inhibition of Abl with STI571 rescued the multi-lobular WAVE2-KD 3D phenotype whereas overexpression of Abl kinase phenocopied the WAVE2-KD phenotype.

**Conclusions:**

The WAVE2 complex regulates breast epithelial morphology by a complex mechanism involving repression of Twist1 expression and Abl kinase activity. These data reveal a critical role for WAVE2 complex in regulation of cellular signaling and epithelial morphogenesis.

## Introduction

Epithelial morphogenesis is a complex process that involves coordination of cellular proliferation, apoptosis, and motility through cell-cell interactions [1]–[4]. Defects in cell-cell interactions can therefore result in disorganization of tissues and hyperplasias, which are known precursors of cancer development. A major type of cell-cell junction that serves both a structural and signaling role is the adherens junction (AJ), for which the minimal functional unit is a dimer of cadherin receptors [3], [4]. Cadherins link adjacent cells via their extracellular domains and coordinate interaction with the actin cytoskeleton through cytoplasmic binding partners called catenins. In the mammary gland, alterations in molecules that regulate AJ formation or signaling have been tied to cancer initiation, progression, and metastasis [5], [6].

A concerted adhesive switch that frequently occurs in aggressive cancers is known as epithelial-mesenchymal transition (EMT). EMT is best understood as a developmental process that contributes to mesoderm formation and neural crest delamination [6], [7]. A central feature of canonical EMT is cadherin switching from E-cadherin to a mesenchymal family member, usually N-cadherin, to accommodate the more dynamic adhesive activity associated with elevated cell motility. EMT is also thought to contribute to a cancer stem cell phenotype [6], [8], [9]. The mechanism(s) by which EMT and EMT-like programs are activated in cancer are poorly understood, but are frequently associated with oncogenic cell signaling and activation of gene expression programs orchestrated by master transcription factors, including TWIST, Snail, Slug, and ZEB1 [6], [7].

Another critical determinant of cellular organization and cell-cell adhesion is assembly and regulation of the actin cytoskeleton. Initiation of AJ formation is dependent on protrusion of branched actin-based lamellipodial as well as unbranched filopodial structures [10]–[18]. In addition, dynamic branched actin has been linked to maintenance of AJs, potentially via regulation of cadherin endocytosis [19]–[22]. The majority of these studies have focused on adhesion formation on 2-dimensional (2D) surfaces. By contrast, the contribution of branched actin regulators to higher-order epithelial tissue structures is poorly understood, in part due to early lethality associated with germline loss of a number of Arp2/3 complex regulators [23]–[30]. Interestingly, a recent paper implicated regulators of actin-rich pseudopodial protrusions in EMT [31], suggesting a dynamic interplay between lamellipodial actin dynamics and signal regulation of EMT.

In this study, we tested the effect of manipulating the branched actin lamellipodia regulator, WAVE2, on 3-dimensional (3D) epithelial morphogenesis by MCF10A mammary epithelial cells. WAVE2-knockdown (KD) cells formed large multilobular mammary acini, likely due to aberrant ongoing proliferation in late-stage acini. However, WAVE2 depletion also led to a partial EMT-like phenotype with elevated expression of Twist1 mRNA and cadherin switching from E-Cadherin to N-Cadherin. Interestingly, the WAVE2-KD 3D morphologic phenotype was fully rescued by KD of Twist1, indicating that activation of an EMT-like program is critical to the WAVE2-KD morphogenesis changes. Moreover, the phenotype was partially rescued by exogenous expression of E-cadherin. Interestingly, Western blot analysis of the Abl substrate CrkL revealed an increase in phosphorylation of the canonical Abl phosphosite Y207 in WAVE2-KD cells, suggesting that the WAVE2-binding partner Abl kinase is overly active in WAVE2-KD cells. In addition, treatment of WAVE2-KD cells with the Abl/c-kit/PDGFR kinase inhibitor STI571 rescued acinar morphology. Finally, the WAVE2-KD multilobular morphology was effectively phenocopied in control cells by exogenous expression of Abl kinase in parental MCF10A cells. Altogether these data are consistent with a critical role for WAVE2 in controlling 3D epithelial morphogenesis via regulation of Abl kinase activity and Twist1 levels.

## Results

**Figure 1:**
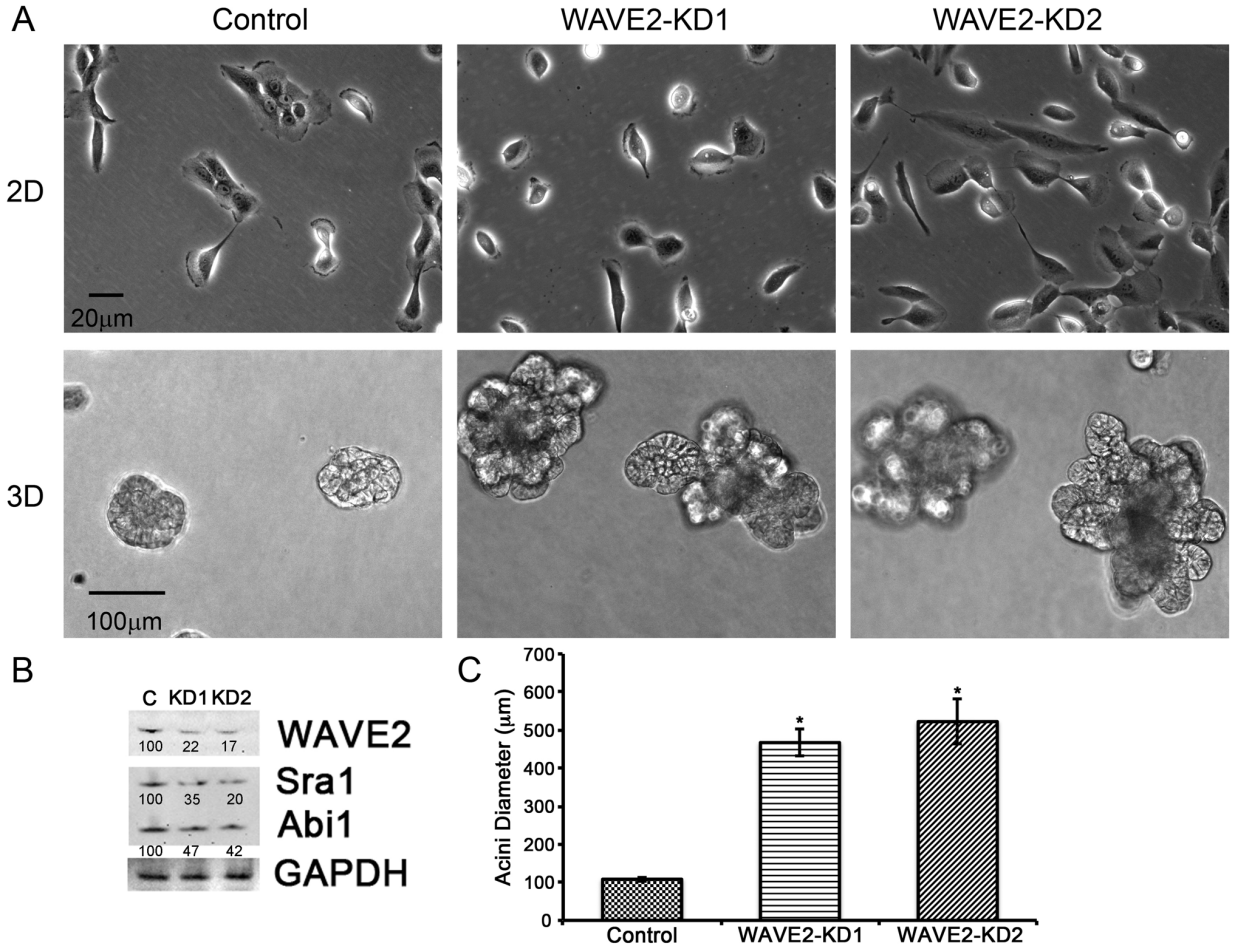
WAVE2 knockdown uniquely results in aberrant 3D morphology of MCF10A epithelial cells. A. Phase contrast images of control and WAVE2-KD cells grown in 2D and 3D cell culture (Day 16). B. Representative Western blots of WAVE2, Abi1 and Sra1 in control and WAVE2-KD cell lines. Numbers below the bands indicate average densitometry values from n = 3 blots normalized to loading control protein levels. Representative GAPDH loading control blot is shown. C. Quantification of average acini diameter. n = 30 acini of each cell type selected from 3 independent experiments, error bars represent standard error of the mean. * indicates p<0.05.

To understand how dynamic membrane protrusions such as lamellipodia contribute to epithelial morphogenesis in 3D, we expressed shRNA against WAVE2 in MCF10A mammary epithelial cells. WAVE2 is an Arp2/3 complex regulator that regulates lamellipodial initiation and stability [28], [29], [32]. Loss of WAVE2 (Figure 1A–B), induced major 3D morphological changes in comparison to control cells, with the formation of large, multi-lobular acini (Figure 1A, C). WAVE2-KD also led to reduced abundance of other WAVE2 complex members, including Sra1 and Abi1 (Figure 1B). The shRNA sequences against WAVE2 contained multiple mismatches with the corresponding sequences of both WAVE1 and WAVE3 (Figure S1A), and the levels of WAVE1 and WAVE3 were not significantly altered in WAVE2-KD cells as measured by qPCR (Figure S1B). In sparse 2D culture, WAVE2-KD cells exhibited reduced cell-cell adhesions and were spread evenly across the plate as single cells, whereas control MCF10A cells formed small colonies (Figure 1A). Consistent with previous findings [28], [29], [32]–[34], lamellipodia and membrane ruffles were largely absent and many cells adopted a rounded morphology in the WAVE2-KD cells (Figure 1A).

**Figure 2:**
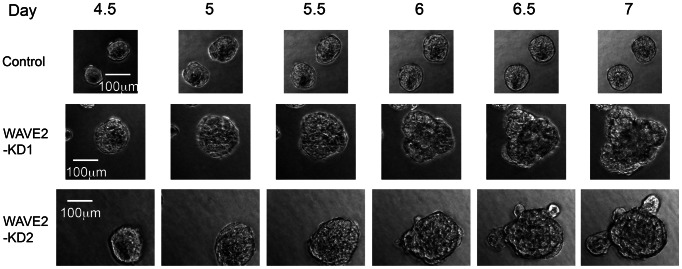
Large multilobular WAVE2-KD acini arise from a single acinar structure. Frames from the Supplemental Movies (S1–S3). Control and WAVE2-KD acini were imaged during 3D culture growth from days 4.5 to 7. Note the formation of lobules from round WAVE2-KD acini during this time period.

We hypothesized that the multi-lobular morphology of WAVE2-KD cells in 3D culture could be a consequence of either the aggregation of multiple acini, or aberrant growth of single acini. To distinguish between those 2 possibilities, long-term live-cell imaging was performed on control and WAVE2-KD acini grown from single cells. Movies were taken over a period of 63 hours, beginning at day 4.5 when both control and WAVE2-KD acini were uniformly round and still proliferating (Figure 2 and Movies S1–S3). Aggregation of acini was not seen in the WAVE2-KD cultures. By contrast, there was aberrant continuation of the proliferative phase such that selective division of a subpopulation of WAVE2-KD cells within each acinus led to the development of lobular “flower-like” structures. By day 5.5, no further increase in the size of control acini was observed whereas proliferation of cells in the WAVE2-KD acini continued for the duration of the movies (7 days post-plating, Figure 2, Movies S1–S3).

**Figure 3:**
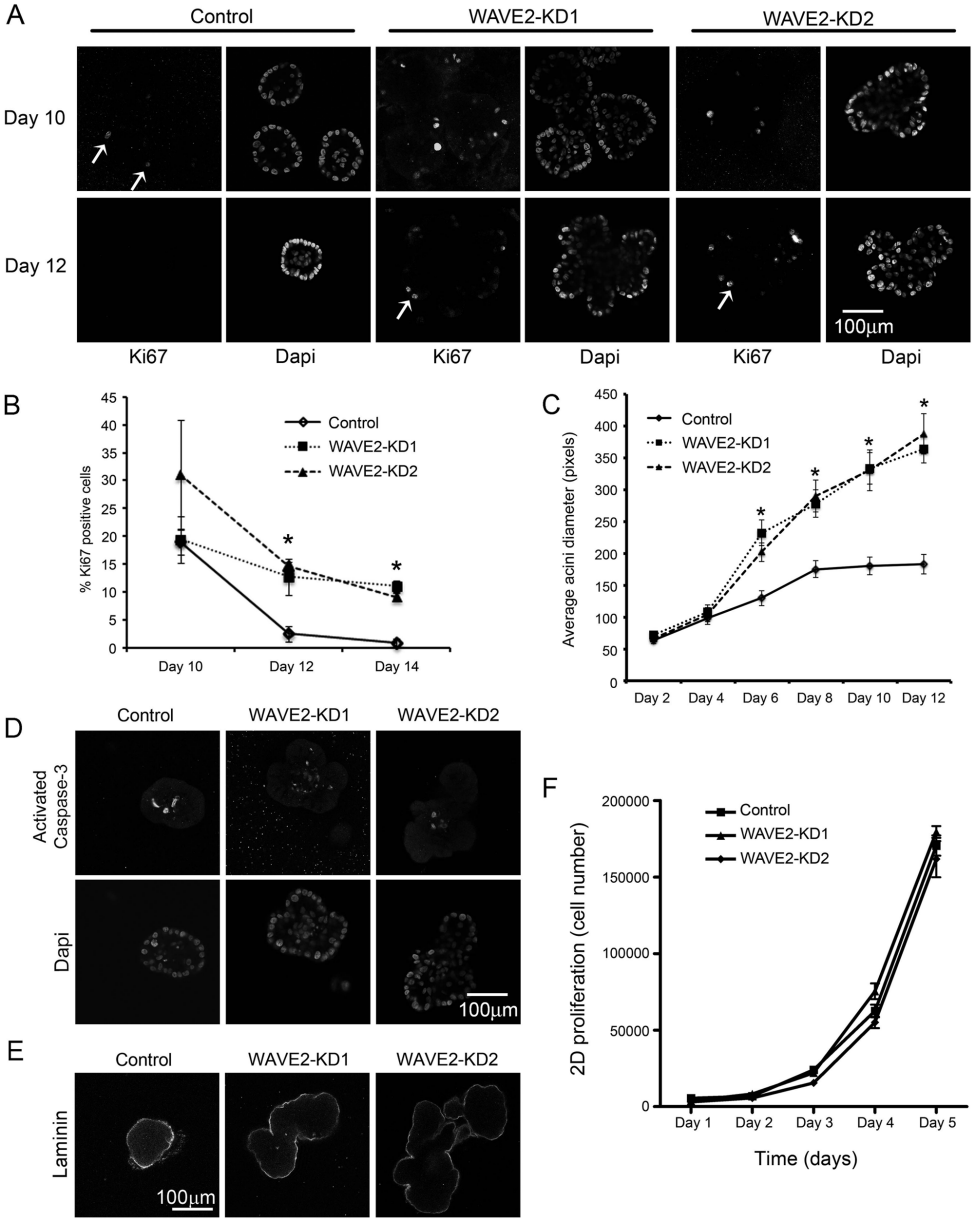
Cellular proliferation is dysregulated in WAVE2-KD cells in 3D culture but not in 2D culture. A. Control and WAVE2-KD acini were immunostained with Ki67 to identify proliferating cells (arrows) and counterstained with DAPI to show total number of nuclei. Single confocal slices through the central region of the acini are shown at Days 10 and 12 of growth. Scale bar 100 µm. B. Quantification of the number of Ki67 positive cells in acini at Days 10, 12 and 14 of growth. Error bars represent standard error of the mean. * indicates p<0.05. C. Quantification of average acini diameter over time. n = 10 acini of each cell type at each time point selected from 3 independent experiments, * indicates p<0.05. D. Control and WAVE2-KD acini were immunostained with activated Caspase-3 to identify apoptotic cells and counterstained with DAPI to show total number of nuclei. Single confocal slices through the central region of the acini are shown at Day 12 of growth. Scale bar 100 µm. E. Control and WAVE2-KD acini were immunostained with laminin-332 to indicate basal polarity. Scale bar 100 µm. F. Cell proliferation of control and WAVE2-KD cells in 2-dimensional cell culture. n =  duplicate wells from 3 independent experiments (6). Error bars represent standard error of the mean. * indicates p<0.05.

To verify that aberrant proliferation was ongoing in late-stage WAVE2-KD 3D cultures, control and WAVE2-KD acini were fixed and immunostained with the proliferative marker Ki67 at various time points. Single confocal images through the middle of the acini were analyzed (Figure 3A). At day 10, low numbers of Ki67-positive cells were seen in the control acini and there were many more Ki67-positive cells in the WAVE2-KD acini. By days 12 and 14, there were essentially no detectable Ki67-positive cells in control acini, whereas Ki67-positive cells were still present in WAVE2-KD acini (Figure 3A, B). The proliferating cells were predominantly localized to the central edge of each lobe, suggesting that this area contributed to expansion of the multilobe structure over time (Figure 3A). Consistent with the ongoing late-stage proliferation in WAVE2-KD cells, there was a significant increase in the size of 6–12 day WAVE2-KD acini compared to controls but no size difference in 2 and 4 day acini (Figure 3C). There was no apparent change in apoptosis of the inner cell mass as activated caspase-3 staining was observed in both control and WAVE2-KD acini and all acini had hollow lumens (Figure 3D) [35]. There was also no apparent defect in cell polarization based on Laminin-332 localization to the basal surface of both control and WAVE2-KD acini (Figure 3E) [35]. Interestingly, there was no difference in the rate of control or WAVE2-KD cell proliferation in 2D cultures (Figure 3F), indicating that the aberrant ongoing proliferation observed in late-stage WAVE2 acini is a 3D context-dependent event.

**Figure 4:**
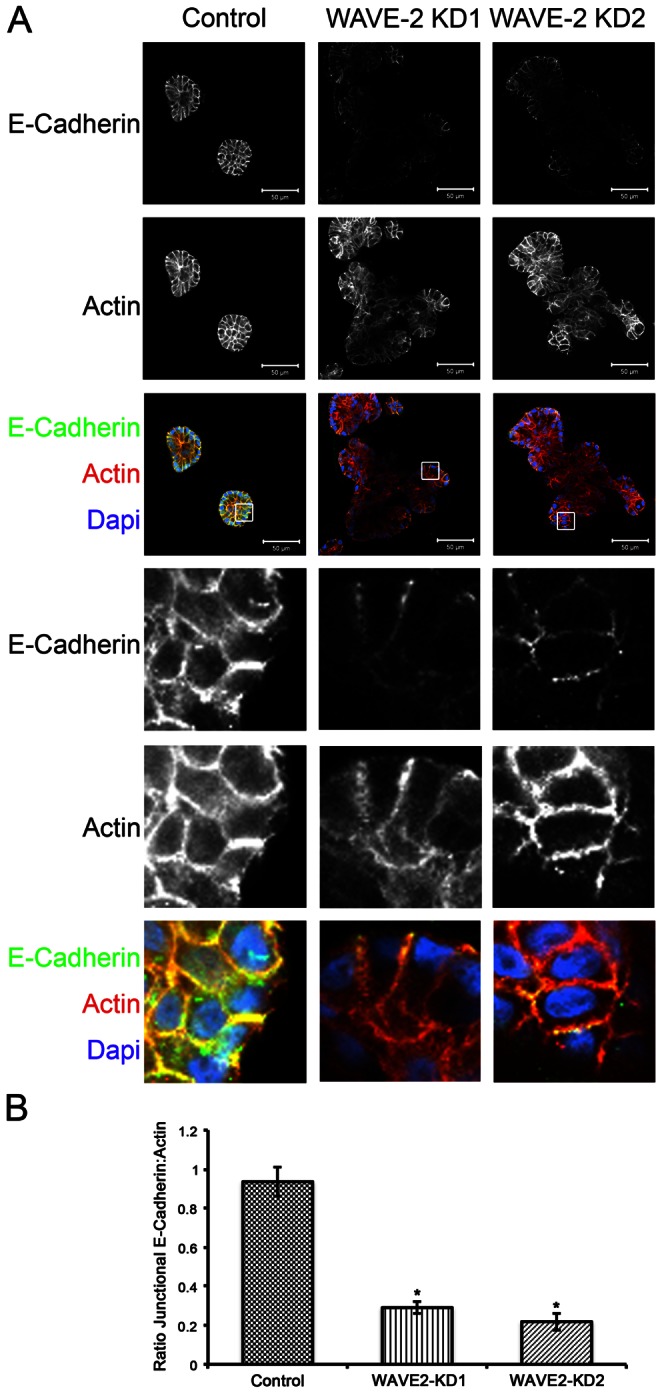
Loss of WAVE2 results in heterogeneous E-Cadherin localization at cell-cell adhesions. A. Confocal images were captured through the center of control and WAVE2-KD acini immunostained for E-Cadherin (green) and counterstained with Alexa-568 phalloidin (red) to label actin filaments and DAPI (blue) to label nuclei. The white boxes in the low power merged images indicate location of the enlarged areas shown below in the lower panels. Note low and heterogeneous E-cadherin staining at actin-positive cell-cell junctions. Scale bar 50 µm. B. Quantification of the ratio of junctional E-Cadherin to actin. n = 50 cell-cell junctions. Error bars represent standard error of the mean, * indicates p<0.05.

Because branched actin assembly is critical for proper function of AJs [15], [19], [22] and AJ formation is enhanced in 3D, we hypothesized that alterations in cell-cell adhesion in WAVE2-KD cells might affect epithelial morphogenesis. To visualize AJs and junctional actin, acini were stained with antibodies against E-Cadherin and Alexa-568 phalloidin (single confocal images from the middle of representative acini shown in Figure 4). Heterogeneous and low-level E-Cadherin expression at cell-cell junctions was seen in WAVE2-KD acini whereas control acini had comparatively strong and contiguous E-Cadherin expression at cell-cell junctions (Figure 4A). Actin at the cell-cell junctions was surprisingly still present and localized correctly in WAVE2-KD cells, despite the relative lack of E-cadherin. Quantification of the ratio of E-Cadherin to actin at the cell-cell junctions showed an average 3-fold decrease of E-Cadherin in the WAVE2-KD knockdown cells compared to control cells (Figure 4B). The heterogenous staining and overall decrease in the amount of E-Cadherin at cell-cell adhesions in WAVE2-KD cells was also observed in 2D cultures (Figure S2), consistent with the reduced 2D colony formation compared to control cells (Figure 1B). The loss of WAVE2 affected cell-cell adhesive strength as determined by a hanging drop assay. In this assay, cells are suspended in media so that they form aggregates and cell-cell junctions rather than adhering to an extracellular matrix substrate. The aggregated cells are then mechanically disrupted by a standardized pipetting regime; dispersion indicates weak cell-cell junctions [36]. WAVE2-KD aggregates were much more easily dispersed into single cells than control aggregates, indicating loss of cell-cell adhesive strength (Figure S3).

**Figure 5:**
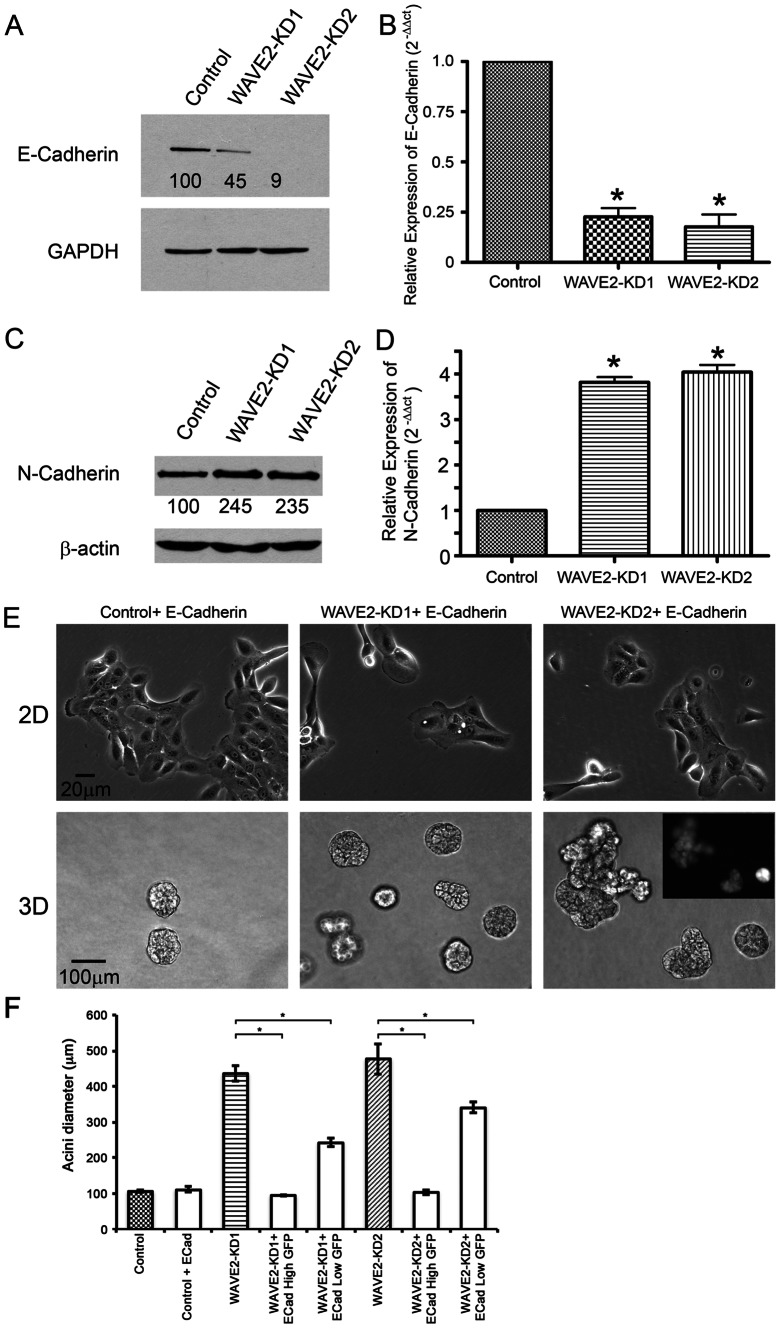
E-Cadherin is downregulated at the RNA level in WAVE2-KD cells and contributes to the aberrant WAVE2-KD acinus morphology. A. Western blot of E-Cadherin levels within control and WAVE2-KD cells. GAPDH levels are shown as a loading control. Numbers below the bands indicate average densitometry values from n = 3 blots normalized to control protein levels. B. Quantitative real-time PCR data of E-Cadherin expression within control and WAVE2-KD cells. n = 3, *p<0.05, compared to control. C. Western blot analysis of N-Cadherin protein levels in control and WAVE2-KD cells. β-actin levels are shown as a loading control. Numbers below the bands indicate average densitometry values from n = 3 blots normalized to control protein levels. D. Quantitative real-time PCR data of N-Cadherin expression in control and WAVE2-KD cells. n = 3, *p<0.05, compared to control. E. Representative phase contrast images of control + E-Cadherin and WAVE2-KD + E-Cadherin cells in 2D and acini grown from those cells in 3D (Day 16). The fluorescence image inset shows GFP levels within the E-Cadherin rescued WAVE2-KD2 acini which is indicative of E-Cadherin levels within the acini. F. Quantification of the average acini diameter of control and WAVE2-KD acini expressing exogenous E-Cadherin. The WAVE2-KD acini were divided into 2 groups based on GFP expression and quantified separately. n = 20 acini of each cell type selected from 3 independent experiments, error bars represent standard error of the mean. * indicates p<0.05.

Because WAVE2-KD cells exhibited heterogeneous and apparent low-level staining of E-cadherin at AJ in both 2D and 3D culture, we hypothesized that the whole cell abundance of E-cadherin might be reduced. Western blot analysis of whole cell lysates from 2D culture demonstrated decreased levels of E-Cadherin in WAVE2-KD cells compared to control cells (Figure 5A). E-Cadherin is known to be degraded in response to its removal from the cell-cell junction; alternatively, levels of E-Cadherin can be transcriptionally regulated [37]. To determine if the mechanism by which E-Cadherin was downregulated in response to loss of WAVE2 was transcriptionally based, we performed quantitative PCR using E-Cadherin specific primers. Surprisingly, we found that there was a 75% decrease in the relative expression of E-Cadherin mRNA in WAVE2-KD cells compared to control cells (Figure 5B). Since decreased transcription of E-cadherin is often a consequence of EMT and occurs in association with increases in N-cadherin expression, we also tested whether N-cadherin mRNA levels were altered. Indeed, consistent with an EMT-like cadherin switch, N-cadherin expression was increased at both the mRNA and protein level (Figure 5C, D). Localization of N-Cadherin was also enhanced at cell-cell adhesions in WAVE2-KD cells compared to control cells grown in monolayer culture (Figure S4). Beta-catenin localized to cell junctions in control and WAVE2-KD cells in both 2D and 3D culture (Figure S5).

To test whether decreased E-cadherin expression could account for the formation of multi-lobular acini in WAVE2-KD cells, E-cadherin was re-expressed along with GFP under the control of a common promoter and with an internal ribosomal entry site (IRES) so that the GFP level within a cell could be used to assess E-Cadherin levels in individual cells. In 2D, re-expression of E-cadherin in WAVE2-KD cells fully rescued the cell-cell adhesion phenotype, with similar colony formation as control cells (Figure 5E, compare to KD phenotype in Figure 1E). In 3D cultures, however, restoration of normal morphology was observed in many but not all acini, dependent on the level of E-Cadherin expression. Note that since each acinus grows from a single cell, each acinus is clonal and presumably has uniform GFP, E-cadherin and WAVE2 expression. Thus, as shown in the inset in Figure 5E, only WAVE2-KD acini expressing high levels of GFP and therefore presumably high levels of E-Cadherin were morphologically rescued. To quantitate these results, the acini were split into 2 groups dependent on GFP expression and the average diameter of the acini was measured. High GFP expressing acini had an average diameter similar to that of control cells, whereas low GFP expressing acini were significantly larger (Figure 5F).

**Figure 6:**
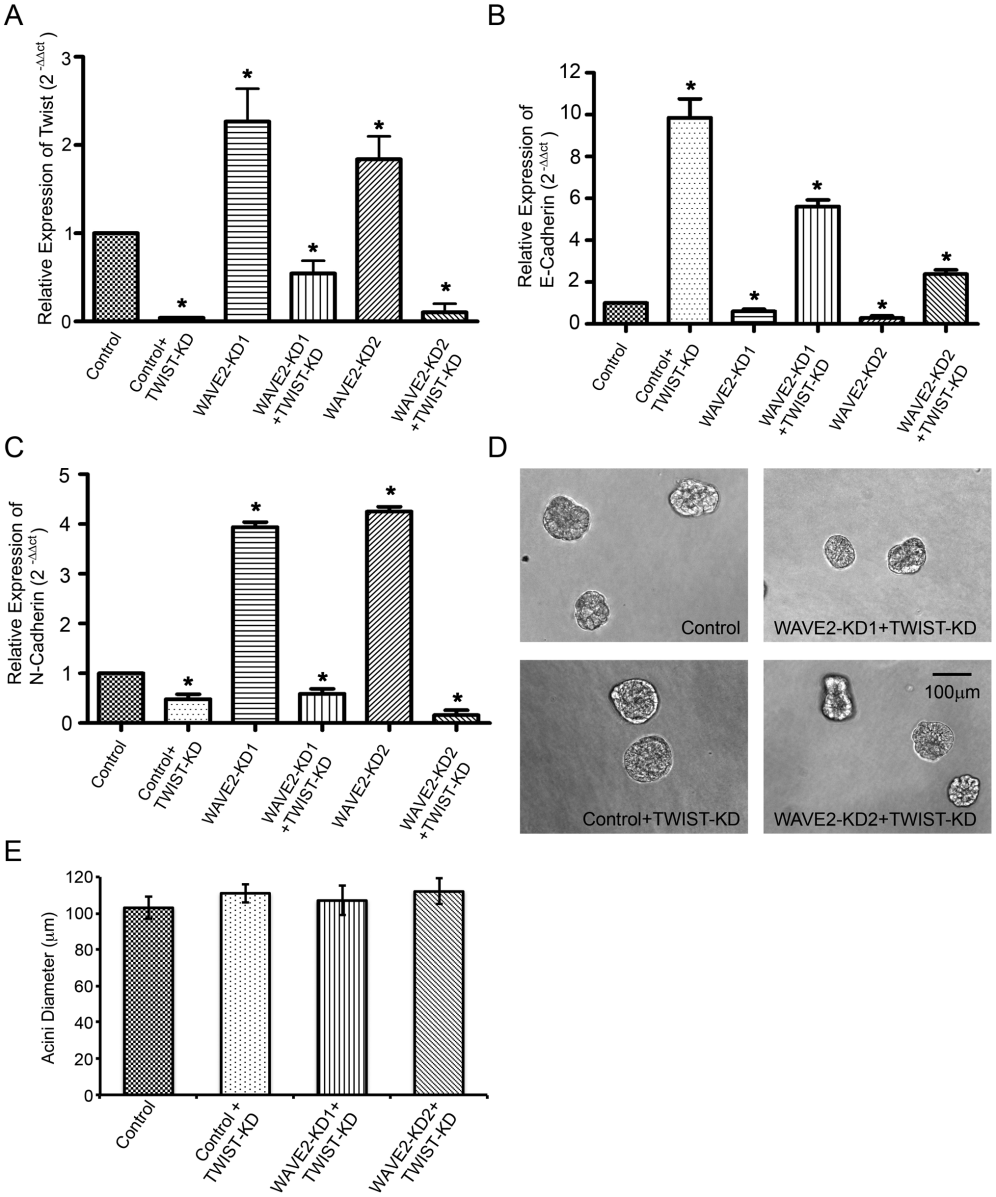
WAVE2 functions upstream of Twist1 to control breast epithelial 3D morphology. A. Quantitative real-time PCR data of Twist1 levels within control, WAVE2-KD cells +/− Twist1-KD. B. Quantitative real-time PCR data of E-Cadherin expression in control and WAVE2-KD cells as well as the respective Twist1-KD cells. C. Quantitative real-time PCR data of N-Cadherin expression in control and WAVE2-KD cells as well as the respective Twist1-KD cells. n = 3, * indicates p<0.05, compared to control. D. Phase contrast images of acini grown from control cells, control + Twist1-KD, and WAVE2-KD + Twist1-KD cells. E. Quantification of average acini diameter of Twist1 knockdown acini. n = 30 acini of each cell type selected from 3 independent experiments, error bars represent standard error of the mean. Values were not significantly different from control acini.

The mechanisms by which the E-Cadherin gene is transcriptionally regulated include alterations in transcription factor activity and epigenetic modification [38]–[42]. Since our data indicated that WAVE2 loss led to a coordinated decrease in E-cadherin and increase in N-Cadherin mRNA levels, we analyzed the expression of three EMT-associated transcription factors known to drive cadherin switching in breast cancer cell lines, Slug, Snail and Twist1 [40]–[42]. Slug and Snail were undetectable in both control and WAVE2-KD MCF10A cells by quantitative PCR (data not shown); however, expression of Twist1 was upregulated 2-fold in the WAVE2-KD cells compared to control cells as determined by quantitative PCR (Figure 6A). To determine if the mechanism by which WAVE2 regulates the cadherin mRNA abundance and 3D morphology is through upregulation of Twist1 expression, we knocked down Twist1 in both control and WAVE2-KD cells using retrovirally transduced shRNA (Figure 6A). As expected, knockdown of Twist1 led to an increase in E-cadherin and a decrease in N-cadherin mRNA abundance in both control and WAVE2-KD cells (Figure 6B, C), as well as to an increase in the intensity of E-cadherin at cell-cell junctions (Figure S6). Interestingly, when grown in 3D culture, the knockdown of Twist1 in the WAVE2-KD cells (WAVE2-KD + Twist1 KD) completely reversed the phenotype of WAVE2 knockdown, with the WAVE2-KD + Twist1 KD cell acini being morphologically indistinguishable from control acini (Figure 6D, E, compare to WAVE2-KD in Figure 1E). These data indicate that Twist1 functions downstream of WAVE2 complex to control cadherin RNA abundance and 3D morphogenesis.

As sequence analysis of WAVE2 did not reveal any direct DNA binding domains, it seemed likely that the regulation of Twist1 and cadherin mRNA expression was indirect and potentially downstream of signaling proteins. We therefore searched for known binding partners of WAVE2 complex that have been associated with EMT. A likely intermediary was the WAVE2-binding partner Abl kinase [43], [44], which was shown in different contexts to either promote [45] or inhibit [46] EMT in cultured cell lines. Abl kinase has been shown to bind and activate WAVE2 both via direct interactions with the WAVE2 polyproline domain as well as indirectly through the WAVE complex protein Abi1 [43], [44]. In addition, Drosophila Abi has been shown to genetically antagonize Abl activity in synaptogenesis, axonogenesis, and cellular actin distribution [47]. Thus, we hypothesized that loss of WAVE2 complex members might affect the levels and/or activity of Abl kinase leading to the observed morphological changes.

**Figure 7:**
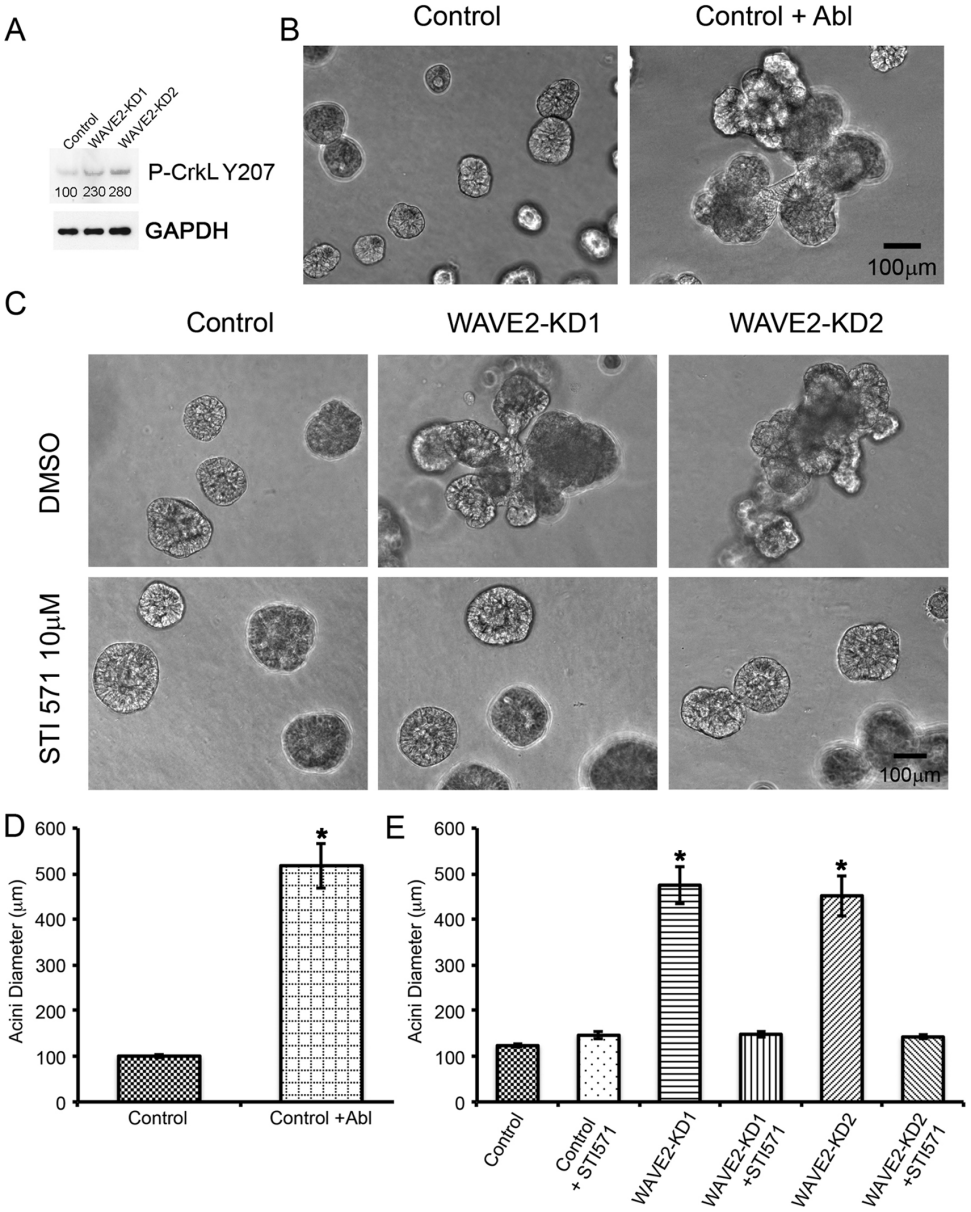
Abl kinase-WAVE2 interactions are critical for regulation of epithelial morphology. A. Western blot of phosphorylated CrkL (Y207) in control and WAVE2 knockdown cells and a western blot of GAPDH as a loading control. Numbers below the bands indicate average densitometry values of P-CrkL from n = 3 blots. B. Phase contrast images of acini from control MCF10A cells or MCF10A cells transfected with full length Abl kinase. C. Phase contrast images of control and WAVE2-KD acini that were grown in the presence of 10 µM STI571 or vehicle control. D. Quantification of average acini diameter of control and Abl overexpressing acini. n = 20 acini of each cell type selected from 3 independent experiments, error bars represent standard error of the mean. * indicates p<0.05. E. Quantification of average acini diameter of STI571 treated acini compared to untreated acini. n = 30 acini of each cell type selected from 3 independent experiments, error bars represent standard error of the mean. * indicates p<0.05.

To test whether Abl kinase activity was altered in WAVE2-KD cells, we performed Western blot analysis of the phosphorylation status of the Abl substrate CrkL [48]–[50]. Indeed, we found that WAVE2-KD cells exhibited increased abundance of phosphorylated CrkL (Y207) (Figure 7A, Figure S7), suggesting that Abl kinase activity is elevated in WAVE2-KD cells. Likewise, there was an apparent increase in the intensity and focal adhesion localization of phospho-CrkL immunofluorescent staining in WAVE2-KD cells compared to controls (Figure S7). We also performed immunofluorescent staining of Abl kinase and found a slight decrease in Abl localization at cell-cell adhesions in WAVE2-KD cells (arrowheads in Figure S8), compared to control cells (arrows in Figure S8); however there was no difference in cytoplasmic localization (Figure S8). To determine whether alteration in Abl kinase levels and/or activity could affect 3D morphogenesis, we overexpressed full length Abl kinase in control cells. Interestingly, similar to WAVE2-KD, overexpression of wt Abl kinase resulted in the formation of large multi-lobular 3D structures (Figure 7B). We also cultured control and WAVE2-KD cells in the presence of the Abl kinase inhibitor STI571 for 16–20 days. Interestingly, 10 µM STI571 completely reversed the WAVE2-KD 3D morphological phenotype and led to normally formed small round mammary acini (Figure 7C). As with WAVE2-KD, treatment with STI571 did not affect cellular localization of beta-catenin (Figure S5A). In addition to Abl kinase, STI571 is known to target c-kit [51]and PDGFR [52]; however, our findings that endogenous phosphorylation of the Abl substrate CrkL is elevated in WAVE2-KD cells and that overexpression of Abl leads to an identical 3D morphogenesis defect as WAVE2-KD suggests that Abl is the likely target of STI571 that controls acinar morphogenesis.

## Discussion

The work presented here indicates that the WAVE2 complex plays an important and complex role in 3D morphogenesis of breast epithelial cells, by suppressing proliferation of late stage acini. We also identified a partial EMT-like phenotype in WAVE2-KD cells, with elevation in Twist1 levels and cadherin switching. Twist1 was linked to the morphogenesis defects, as knockdown of Twist1 in WAVE2-KD cells fully rescued the acinar development phenotype. Finally, we showed that the WAVE2 complex binding partner Abl also regulates mammary acinus development and is overly active in WAVE2-KD cells. Altogether, these data present a complex role for the WAVE2 complex in controlling breast epithelial morphogenesis through Abl kinase and the transcription factor Twist1.

Abl kinases have been shown to activate WAVE2 directly through phosphorylation [43], [44] and indirectly through Rac [53]. The WAVE2 complex and Abl have also been reported to co-regulate a variety of functions in diverse cell types, including pathogen entry into cells [54], [55], T-cell signaling [56], and induction of membrane ruffling and cell spreading [57]. Our data indicate that WAVE2 complex not only acts downstream of Abl kinase but also upstream to regulate Abl kinase activity, as indicated by the increased phosphorylation of the Abl substrate CrkL. We also found that incubation of cells in 3D culture with the Abl/c-kit/PDGFR kinase inhibitor STI571 fully rescues the morphology defect of WAVE2-KD cells. Finally, Abl overexpression induces a multi-lobular acinus morphology that phenocopies that of WAVE2-KD cells. Taken together, our data are consistent with a model in which the WAVE2 complex can regulate Abl kinase activity and downstream morphogenesis events.

Both WAVE2 itself and the WAVE2 complex member Abi1 are known to bind directly to Abl kinase and both were downregulated in WAVE2-KD cells. It may be difficult to distinguish between effects of WAVE2 and Abi1, since Abi1 abundance depends on WAVE2 (Figure 1) and vice versa [58], [59]. Abl kinase has been previously shown to affect epithelial morphogenesis, especially in Drosophila, through the regulation of actin assembly [60]–[63]. However, it is also possible that transcription could occur downstream of Abl activity to regulate adherens junction composition either separately or in conjunction with direct effects on actin assembly.

A novel finding of this study was that the transcription factor Twist1 was upregulated in WAVE2-KD cells and mediated both cadherin switching and epithelial morphogenesis changes. At this point, the mechanism by which the WAVE2 complex regulates Twist1 regulation is unknown. Allington *et al.* found that Abl suppressed Twist1 expression and cadherin switching downstream of TGFβ stimulation by an unknown mechanism [46]. In the context of WAVE2-KD, our morphogenesis results suggest a different relationship in which deregulation of Abl kinase activity may enhance Twist1 expression. A potential experiment that could test the relationship of Twist1 expression to Abl signaling in our system is to analyze Twist1 RNA levels in Abl-KD or STI571-treated control and WAVE2-KD cells. Another important experiment will be to determine if KD of Twist1 can rescue the morphogenesis defect of Abl-overexpressing cells. Even so, it would be unclear exactly how Twist1 could be regulated downstream of Abl kinase. These are important topics for future investigation and could lead to identification of novel mechanisms for induction of EMT and/or epithelial morphogenesis phenotypes.

An interesting finding from our study was that WAVE2-KD led to aberrant ongoing proliferation in a 3D environment but not in 2D. One difference between 3D acinar structure and 2D tissue culture conditions is that cell-cell junctions are much more highly developed and occupy a greater proportion of the plasma membrane in 3D. Thus, one possible explanation for the defective repression of late-stage proliferation in WAVE2-KD MCF10A cells is that altered AJ signaling occurred due to the relative loss of E-cadherin and the increase in N-cadherin at cell-cell junctions. Our finding that the WAVE2-KD acinar morphogenesis phenotype could be fully rescued by KD of Twist1 along with reversal of cadherin switching or partially rescued by re-expression of E-cadherin supports this model. Another possibility is that not only cadherins but also other Twist1 targets are involved in promoting the observed morphological changes in WAVE2-KD acini. For example, Twist and other EMT-promoting transcription factors are known to be critical for branching morphogenesis by promoting not only cadherin switching but also invasion of the surrounding matrix [40], [64]–[66]. Enhanced invasiveness combined with the switch to a mesenchymal cadherin might facilitate outgrowth of acinar lobules that then promotes the observed increase in late-stage proliferation.

Cell-cell adhesion formation and maintenance is a complex process, involving both dynamic actin and junctional signaling proteins [3]. WAVE2 and WAVE2 complex members have previously been shown to mediate actin nucleation at cell-cell adhesions and formation of AJs in 2D culture [18], [22], [67], [68]. However, in 3D culture, we found that there was little effect of WAVE2 expression on junctional actin accumulation; instead, E-cadherin levels were strongly regulated and morphogenesis defects could be rescued by increasing expression of E-cadherin or knock-down of TWIST1. Our unexpected finding of transcriptional regulation of E-cadherin and TWIST1 along with deregulation of Abl kinase activity indicates that regulation of AJs by WAVE2 is more complicated than previously appreciated.

In support of our finding that WAVE2 loss is associated with defective epithelial morphogenesis, a recent paper identified that the loss of Cyfip1, a component of the WAVE2 complex that is lost in invasive breast cancers, resulted in aberrant 3D epithelial morphology and defective AJ formation [69]. Similar changes were found with loss of WAVE2 and other WAVE complex members. Our data are consistent with their findings, but identify a potential signaling mechanism underlying the morphogenesis changes. Furthermore, our finding that WAVE2 complex acts to regulate Abl function and Twist levels suggests a potential selective advantage for loss of WAVE2 complex expression in tumors.

## Materials and Methods

### Cell Culture

MCF10A cells were obtained from Dr Joan Brugge (Harvard Medical School, Boston, MA) and both MCF10A cells and virally transduced MCF10A cell lines were cultured as described [70]. Viral transduction of MCF10A cells was as described [33]. 3D acini were grown on a bed of growth factor reduced Matrigel (BD Biosciences) in 8-well chamber slides following the protocol previously described [70].

### Cellular Assays

Hanging drop assays were performed as described [36]. Briefly, a 30 μl drop of cell media containing 0.5×10^5^ cells was placed on the underside of the lid of a 24 well plate. The lid was then inverted over the wells which contained phosphate buffered saline for humidification and incubated overnight. The aggregated cells were then subjected to 20 pipette strokes, then the cellular aggregate/dispersion imaged by brightfield microscopy.

Cellular proliferation: 1×10^4^ cells/well were plated in a 24 well plate. Each day, duplicate wells were trypsinized and the number of cells counted. The experiment was repeated in triplicate and the error bars represent standard error of the mean. Statistical significance was determined by the Student's t-test.

### Immunofluorescence and image quantitation

For all immunofluorescence and phalloidin staining of both 2- and 3-dimensional cultures, the cells/acini were fixed in 4% paraformaldehyde and permeabilised with 0.4% TritonX-100 and mounted in AquaPolymount (Polysciences Inc.). Immunofluorescence staining of 2D cultures and 3D acini were performed as previously described [33], [70]. Widefield fluorescence images were taken on a Nikon TE 2000-E microscope using a 40X Plan Fluor oil immersion lens objective and captured using a Hamamatsu Orca ER camera and Metamorph software (MDS Inc., Toronto, Canada). Confocal images were obtained using a Zeiss inverted LSM510 confocal microscopy system using a 40x Plan-NEOFLUAR (NA 1.3 Oil; DIC lens) objective and a Hamamatsu Orca ER camera.

Quantitation of Acini size: The average diameter of fixed, DAPI stained acini were measured from fluorescence images using ImageJ (version 1.45s). 10 acini of each cell type at each time point were measured and the average diameter (in pixels) presented. The average diameter (µm) of live acini were measured from phase contrast images using ImageJ (version 1.45s). GFP expressing acini were sorted into 2 groups, below 100 and over 100 average pixel intensity then the diameter measured. Junctional intensity: Regions of interest were defined as approximately 1 pixel on either side of the β-actin cell-cell junction localization of a dual stained sample. The ROI was then transferred to the second image and the average fluorescent intensity of E-Cadherin or beta-catenin immunostaining measured using ImageJ (version 1.45s). 50 cell-cell junctions per sample were measured. The error bars represent standard error of the mean. Statistical significance was determined by the Student's t-test.

### Antibodies and Reagents

Antibodies for Western blotting and immunofluorescence were as follows: E-Cadherin, N-Cadherin, (BD Biosciences), WAVE2 (Upstate), Sra-1, Abi-1 (Santa Cruz), Ki-67 (Calbiochem), activated caspase-3, β-actin, P-Tyr 207 CrkL and GAPDH (all from Cell Signaling Technology), Laminin-332 (Chemicon). Anti-rabbit and anti-mouse HRP conjugated secondary antibodies were from GE Healthcare. Anti-mouse and anti-rabbit Alexa-488 or Alexa-568 conjugated secondary antibodies, Alexa-488 phalloidin and Alexa-568 phalloidin were from Invitrogen. STI 571 was obtained from LC Labs.

### Live-Cell Imaging

Acini were grown in 8-well chamber slides and after 4.5 days of culture the media was changed to L15 media with the required supplements to allow growth in a low CO_2_ atmosphere. Cells were imaged on a Nikon TE 2000-E microscope at 37°C in a humidified environment using a 10X Plan Fluor 0.3NA objective and captured using a Hamamatsu Orca ER camera and Metamorph software (MDS Inc., Toronto, Canada) for 2.5 days. Phase contrast images were taken at multiple stage positions every 30 minutes. The individual images are displayed at a frame rate of 30 frames per second in the resultant movies.

### Construction of siRNA and retroviral expression constructs

Sixty four base pair oligonucleotides (IDT) containing the sense and antisense sequences flanking a 6 base hairpin were inserted into the pRS vector (OligoEngine). The sense sequences used were WAVE2-KD1 catacaatacctgtgatac [33], WAVE2-KD2 gaaagataatccaaatcga [71] and Twist aagctgagcaagattcagacc [72]. The E-Cadherin IRES GFP construct has been previously described [73]. The MSCV2.2-based vector pTK209.5 was generated by inserting the 1.4 kb BamHI EcoRI frament containing an IRES-neoR cassette from into the BamHI-EcoRI sites of MSCV2.2. The murine c-abl type IV cDNA was excised from the pPL vector [74] via digestion at the 5′ end with EcoRI, blunting of 5′ end with Klenow, ligation of 5′ end to NotI linkers. The cDNA was subsequently cut with HindIII at the 3′ end, blunted with Klenow, and cut with NotI. The resulting 3.4 fragment containing c-abl was cloned into pTK209.5 that was digested with XhoI, blunted with Klenow fragment, ligated to NotI linkers, and digested with NotI, HpaI and shrimp alkaline phosphatase.

### Quantitative real time PCR

RNA was extracted (RNAeasy, Qiagen) and cDNA generated (iScript reverse transcriptase, Biorad) according to the manufacturer's instructions. Real time PCR was performed using a BioRad iCycler. For each experimental sample, transcript levels were analyzed by the 2^−ΔΔCt^ method. Transcript levels were normalized to GAPDH levels and analysed in triplicate. The primers used for WAVE2 were tctgataccaccaagcccaa (forward) and cacaacatcccgcttctctt (reverse).

All other primers used for QPCR have been previously described (WAVE1, WAVE3 [75], E-Cadherin, hTwist, Slug [76], hGAPDH [77], hβ-actin [78], and Snail [79]. Statistical significance was determined by the Student's t-test.

## Supporting Information

Figure S1 **Knockdown of WAVE2 does not alter WAVE1 or WAVE3 transcript levels.** A. Alignment of WAVE2 specific knockdown oligonucleotide sequences with the corresponding nucleotide sequence of WAVE1 and WAVE3. Asterisks indicate identical nucleotides. B. Quantitative real-time PCR data of WAVE1 and WAVE3 expression in control and WAVE2-KD cells. n = 3. Data were not significantly different.
(TIF)

Figure S2 **WAVE2-KD cells exhibit a decrease in E-Cadherin at cell-cell adhesions.** Control (A–C) and WAVE2-KD (D–F) cells were cultured in monolayer 2D culture before fixation and immunostaining with Alexa-568 phalloidin (A, D, red in merge) or E-Cadherin (B, E, green in merge). Wide-field epifluorescent images are shown. C. Merged images. Nuclei are stained blue with DAPI in the merged images. Scale bar  = 10 µm.
(TIF)

Figure S3 **KD of WAVE2 results in decreased cell-cell adhesive strength.** Hanging drop cell adhesion assays showing representative images both before (“Pre”) and after (“Post”) pipetting from control and WAVE2-KD cell aggregates. Both KD lines showed decreased cell-cell adhesion strength as evidenced by the dispersal into single cells after pipetting (Compare “Post” of KD lines to control). n = 3 independent experiments.
(TIF)

Figure S4 **WAVE2-KD cells exhibit an increase of N-Cadherin localization cell-cell adhesions.** Single confocal images of control and WAVE2-KD cells cultured in monolayer 2D culture after fixation and immunostaining with N-Cadherin. Scale bar  = 10 µm.
(TIF)

Figure S5 **Loss of the WAVE2 complex does not alter β-catenin localization in 2 or 3-dimensional culture.** A. Widefield fluorescence images of β-catenin staining in Control and WAVE-2 KD cells treated with either vehicle control (DMSO) or 10 μm STI571. Scale bar 10 μm. B. Single confocal images taken through the center of the acini of control and WAVE2-KD acini immunostained with β-catenin and stained with Alexa-488 phalloidin. The merged images represent β-catenin (red), phalloidin (green) and DAPI stained nuclei (blue). The white square shows the area enlarged in the far right panels. Scale bars are 50 μm.
(TIF)

Figure S6 **Knockdown of Twist in WAVE2-KD cells restores E-Cadherin to adherens junctions.** Widefield fluorescence images of E-Cadherin immunostaining and Alexa-568 phalloidin staining in A. Control cells. B. WAVE2-KD1 cells. C. WAVE2-KD2 cells. D. Twist1-KD cells. E. WAVE2-KD1/Twist1-KD cells and F. WAVE2-KD2/Twist1-KD cells. The merged images show E-Cadherin (green), phalloidin (red) and DAPI stained nuclei (blue). Note rescue of the low and heterogeneous E-cadherin staining of WAVE2-KD cells in the WAVE2-/Twist1-KD cells. Scale bar 10 µm.
(TIF)

Figure S7 **Localization of P-CrkL (Y207) in control and WAVE2-KD cells in 2D culture.** Widefield fluorescence images of P-CrkL (Y207) immunostaining and Alexa-568 phalloidin staining in control and knockdown WAVE2 cells. The merged images show P-CrkL (Y207) (green), phalloidin (red) and DAPI stained nuclei (blue). The zoomed images in the bottom row are of P-CrkL (Y207) staining from the regions highlighted by the white boxes in the top row. Scale bar 10 µm.
(TIF)

Figure S8 **Localization of Abl in control and WAVE2-KD cells in 2D culture.** Single confocal images of Abl immunostaining and Alexa-568 phalloidin staining in control and WAVE2-KD cells. The merged image represents Abl (green), phalloidin (red) and DAPI stained nuclei (blue). Arrows point to Abl localization at cell-cell adhesions in control cells whereas arrowheads point to decreased Abl localization at cell-cell adhesions in WAVE2-KD cells. Scale bar 10 µm.
(TIF)

Movie S1 **Long term live cell imaging of control acini grown in 3D culture from day 4.5 to day 7.** Movies were created from images taken every 30 minutes over a period of 2.5 days and displayed at 30 fps. The scale bar is indicated in Figure 2.
(AVI)

Movie S2 **Long term live cell imaging of WAVE2-KD1 acini grown in 3D culture from day 4.5 to day 7.** Movies were created from images taken every 30 minutes over a period of 2.5 days and displayed at 30 fps. The scale bar is indicated in Figure 2.
(AVI)

Movie S3 **Long term live cell imaging of WAVE2-KD2 acini grown in 3D culture from day 4.5 to day 7.** Movies were created from images taken every 30 minutes over a period of 2.5 days and displayed at 30 fps. The scale bar is indicated in Figure 2.
(AVI)
