# Supplementary figures and images for: WAVE2 Regulates Epithelial Morphology and Cadherin Isoform Switching through Regulation of Twist and Abl
Source: PLoS One. 2013 May 15;8(5):e64533. doi: 10.1371/journal.pone.0064533 (PMC3654908; doi:10.1371/journal.pone.0064533)

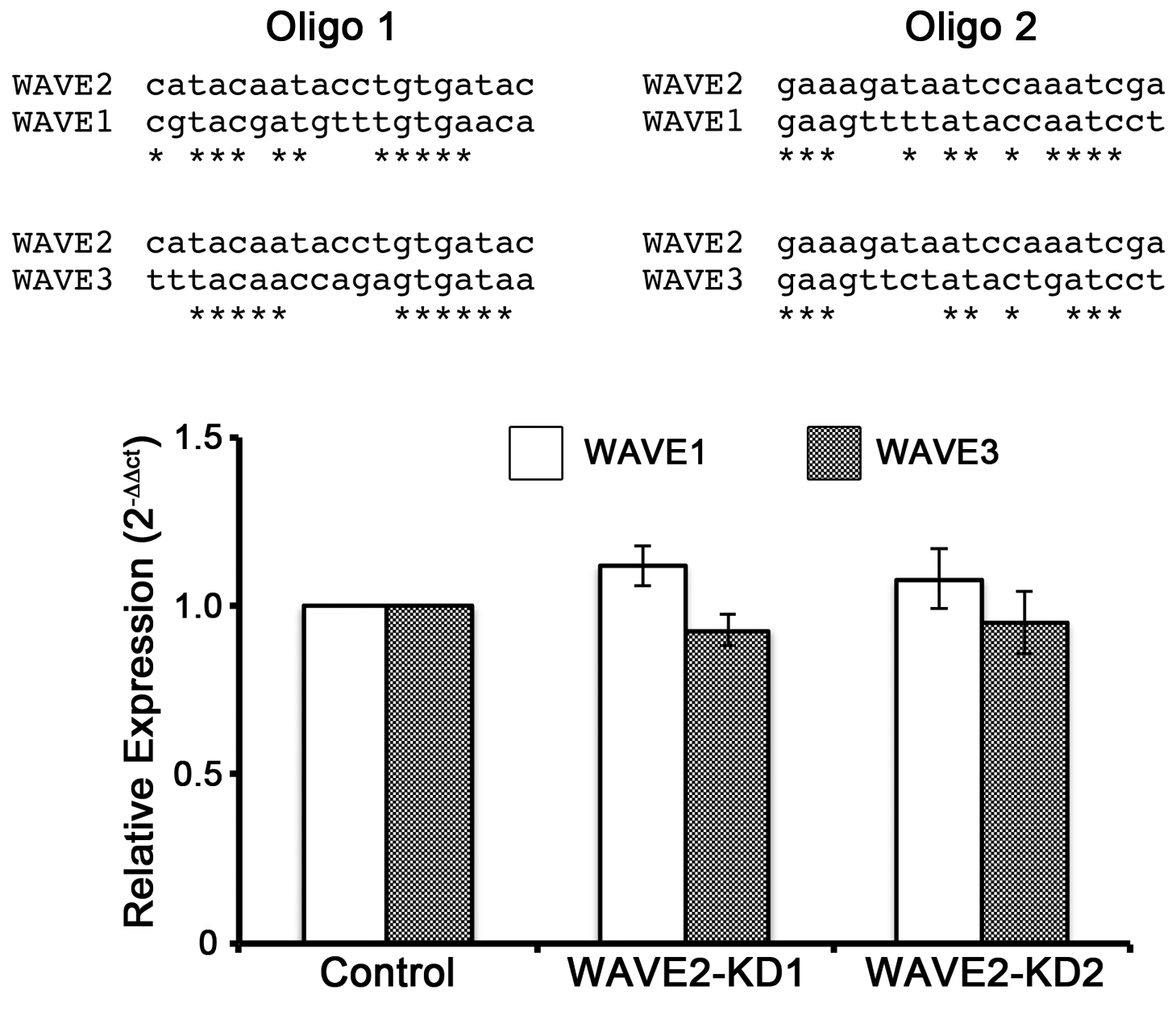

Supplement: Figure S1 — Knockdown of WAVE2 does not alter WAVE1 or WAVE3 transcript levels. A. Alignment of WAVE2 specific knockdown oligonucleotide sequences with the corresponding nucleotide sequence of WAVE1 and WAVE3. Asterisks indicate identical nucleotides. B. Quantitative real-time PCR data of WAVE1 and WAVE3 expression in control and WAVE2-KD cells. n = 3. Data were not significantly different. (TIF) [file pone.0064533.s001.tif]

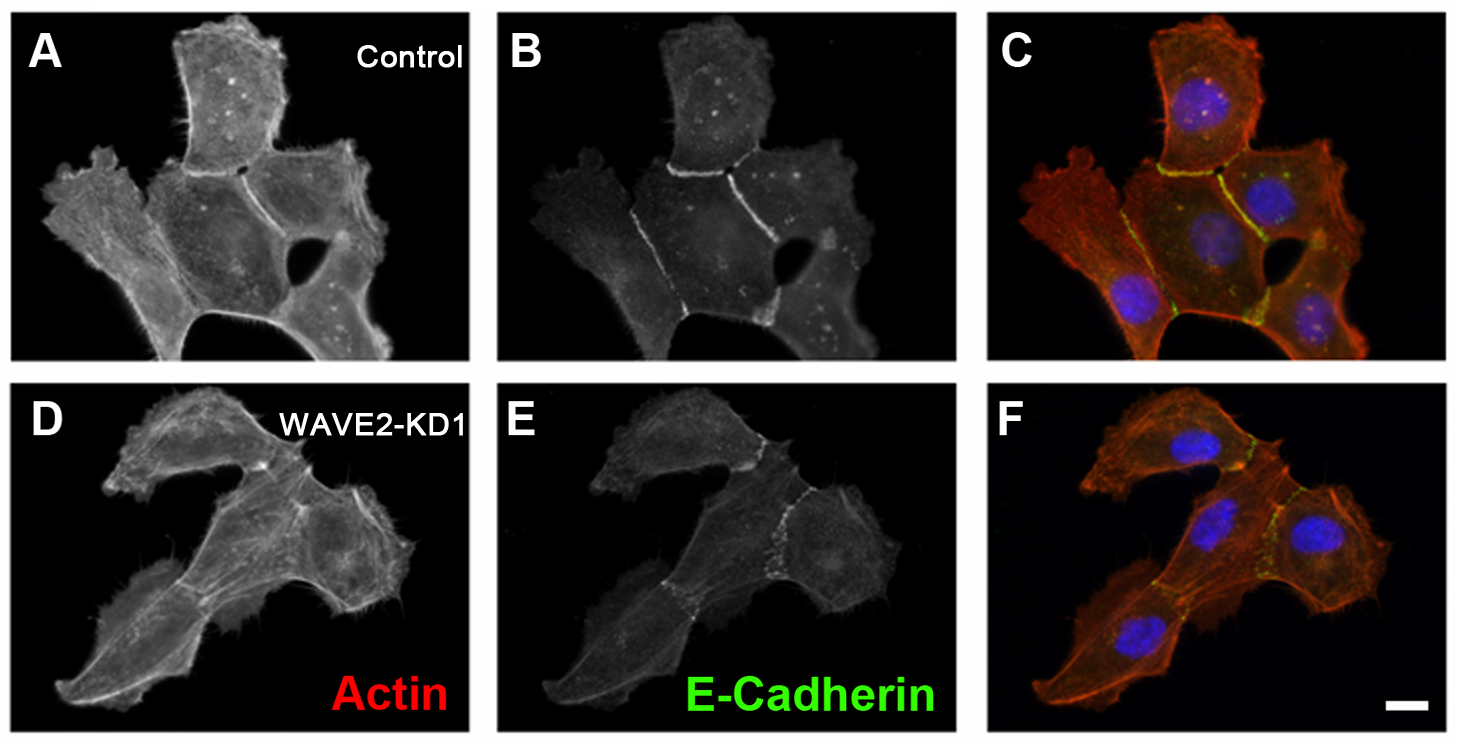

Supplement: Figure S2 — WAVE2-KD cells exhibit a decrease in E-Cadherin at cell-cell adhesions. Control (A–C) and WAVE2-KD (D–F) cells were cultured in monolayer 2D culture before fixation and immunostaining with Alexa-568 phalloidin (A, D, red in merge) or E-Cadherin (B, E, green in merge). Wide-field epifluorescent images are shown. C. Merged images. Nuclei are stained blue with DAPI in the merged images. Scale bar = 10 µm. (TIF) [file pone.0064533.s002.tif]

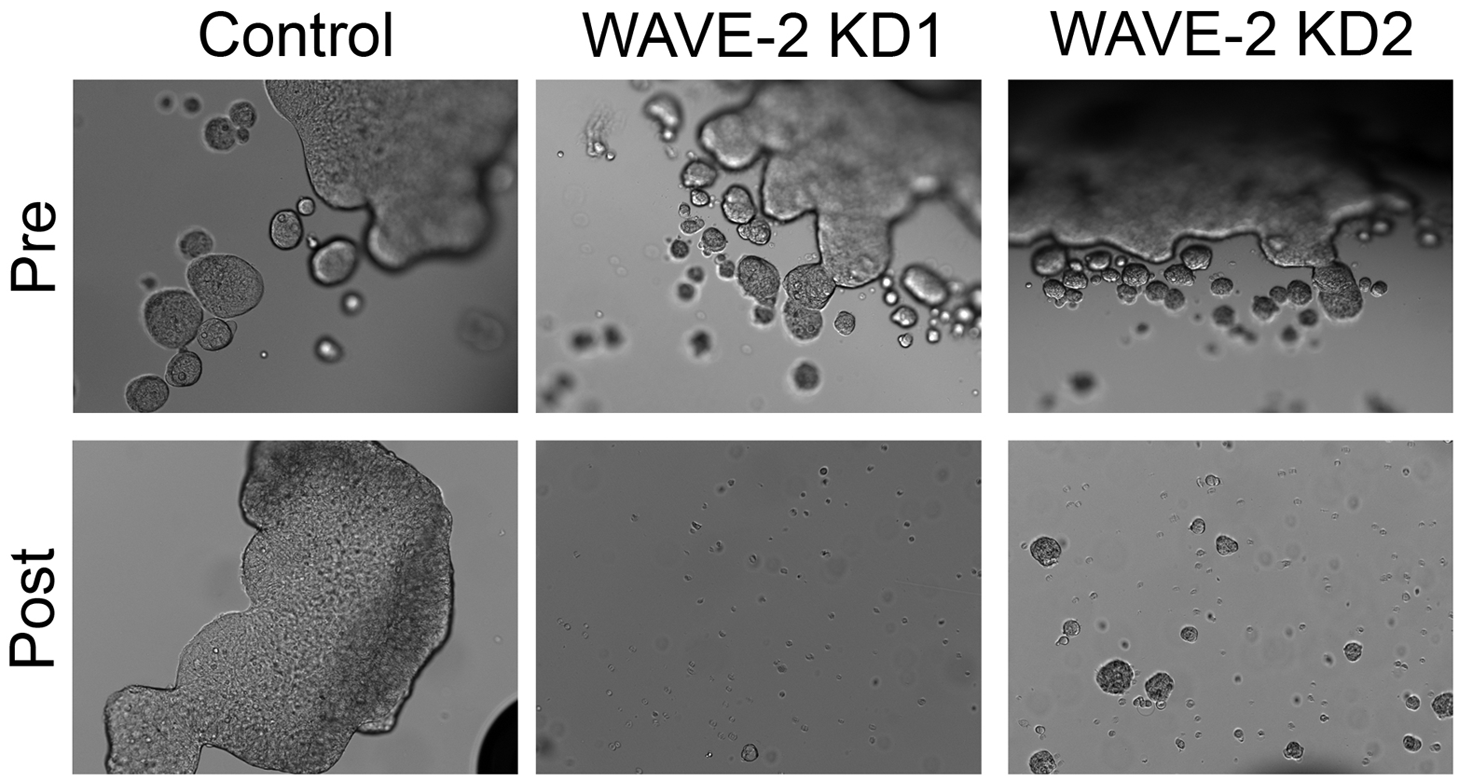

Supplement: Figure S3 — KD of WAVE2 results in decreased cell-cell adhesive strength. Hanging drop cell adhesion assays showing representative images both before (“Pre”) and after (“Post”) pipetting from control and WAVE2-KD cell aggregates. Both KD lines showed decreased cell-cell adhesion strength as evidenced by the dispersal into single cells after pipetting (Compare “Post” of KD lines to control). n = 3 independent experiments. (TIF) [file pone.0064533.s003.tif]

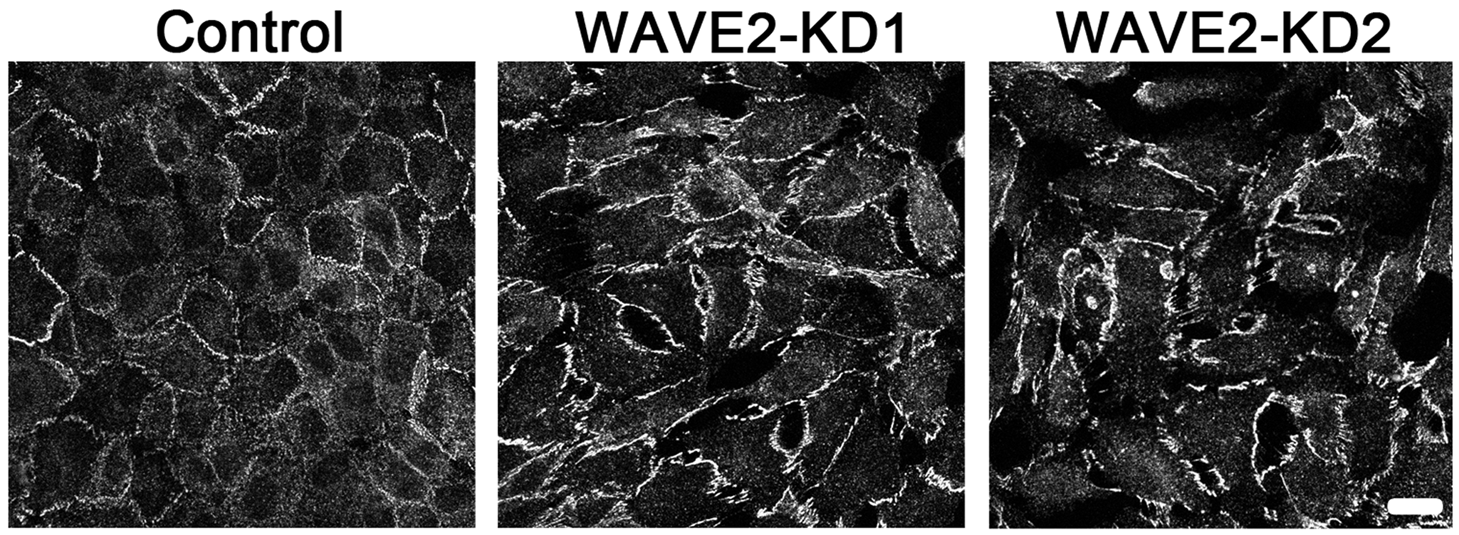

Supplement: Figure S4 — WAVE2-KD cells exhibit an increase of N-Cadherin localization cell-cell adhesions. Single confocal images of control and WAVE2-KD cells cultured in monolayer 2D culture after fixation and immunostaining with N-Cadherin. Scale bar = 10 µm. (TIF) [file pone.0064533.s004.tif]

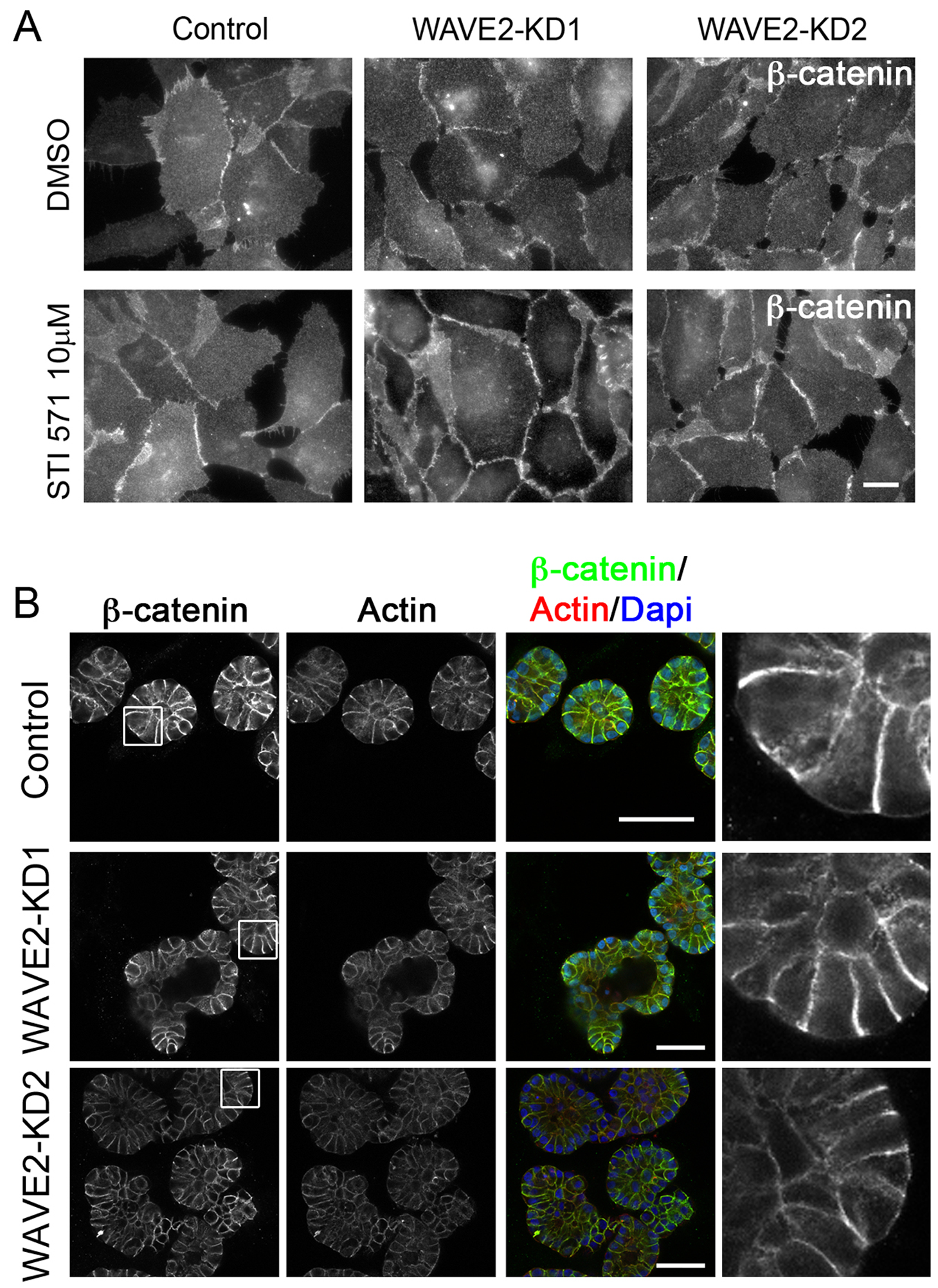

Supplement: Figure S5 — Loss of the WAVE2 complex does not alter β-catenin localization in 2 or 3-dimensional culture. A. Widefield fluorescence images of β-catenin staining in Control and WAVE-2 KD cells treated with either vehicle control (DMSO) or 10 μm STI571. Scale bar 10 μm. B. Single confocal images taken through the center of the acini of control and WAVE2-KD acini immunostained with β-catenin and stained with Alexa-488 phalloidin. The merged images represent β-catenin (red), phalloidin (green) and DAPI stained nuclei (blue). The white square shows the area enlarged in the far right panels. Scale bars are 50 μm. (TIF) [file pone.0064533.s005.tif]

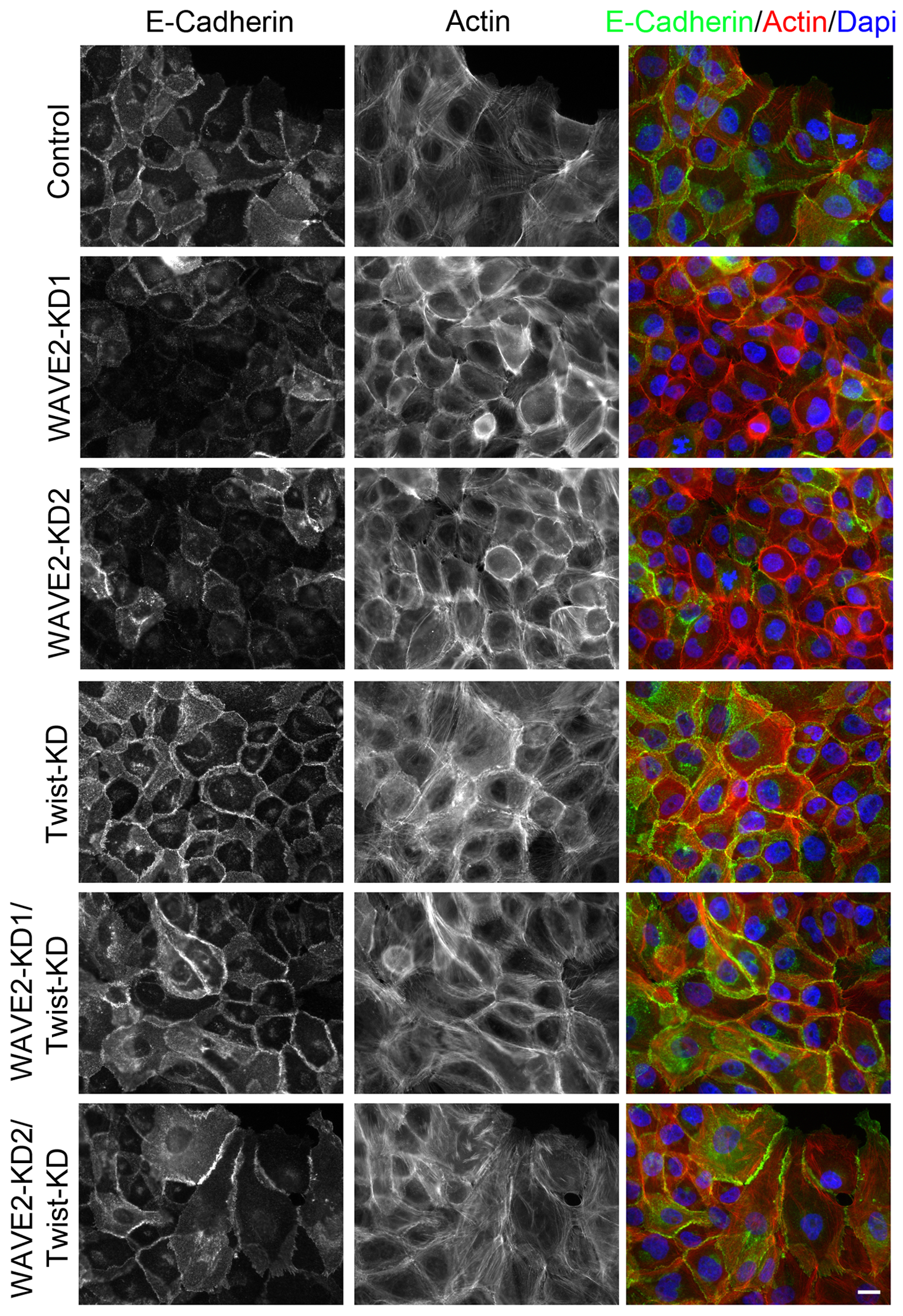

Supplement: Figure S6 — Knockdown of Twist in WAVE2-KD cells restores E-Cadherin to adherens junctions. Widefield fluorescence images of E-Cadherin immunostaining and Alexa-568 phalloidin staining in A. Control cells. B. WAVE2-KD1 cells. C. WAVE2-KD2 cells. D. Twist1-KD cells. E. WAVE2-KD1/Twist1-KD cells and F. WAVE2-KD2/Twist1-KD cells. The merged images show E-Cadherin (green), phalloidin (red) and DAPI stained nuclei (blue). Note rescue of the low and heterogeneous E-cadherin staining of WAVE2-KD cells in the WAVE2-/Twist1-KD cells. Scale bar 10 µm. (TIF) [file pone.0064533.s006.tif]

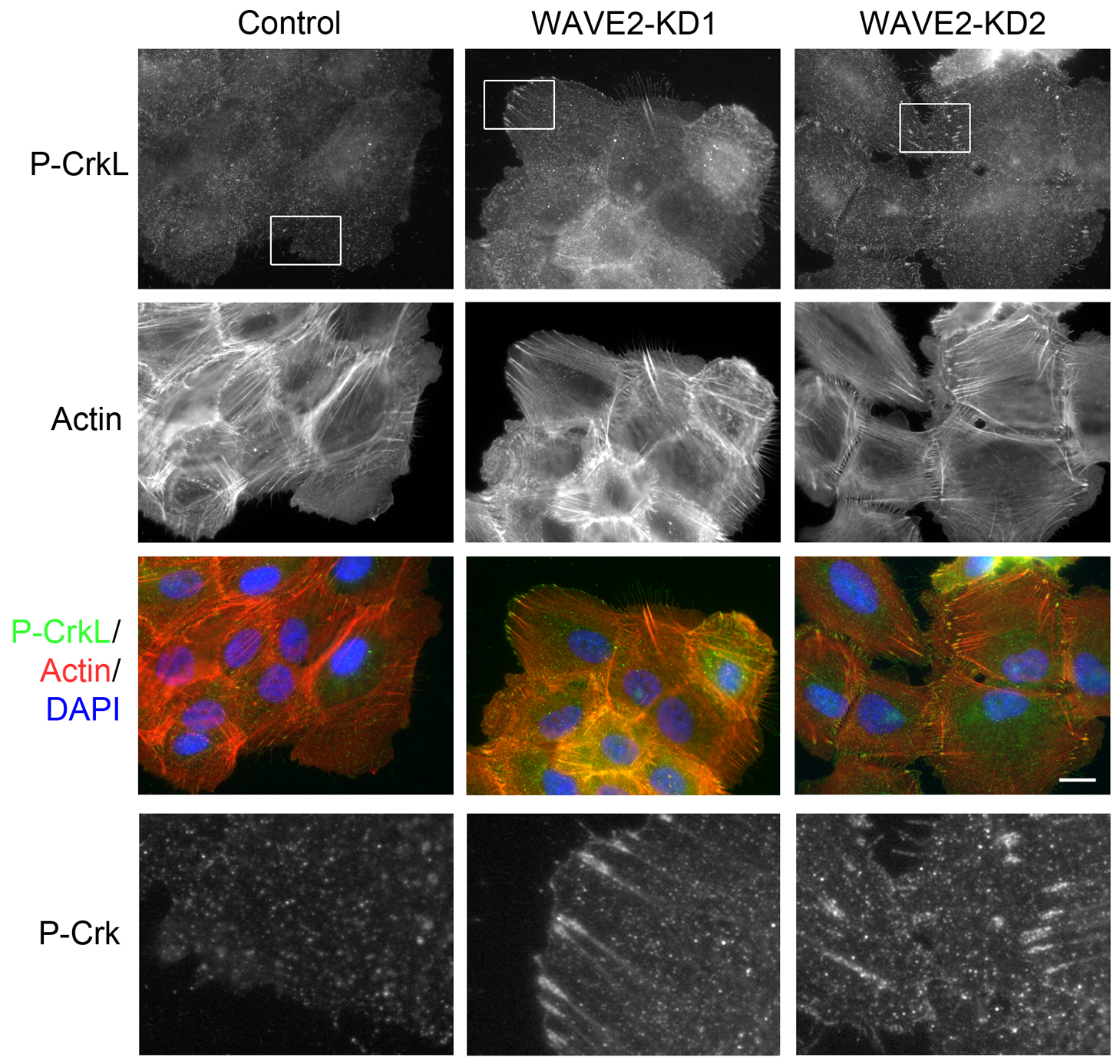

Supplement: Figure S7 — Localization of P-CrkL (Y207) in control and WAVE2-KD cells in 2D culture. Widefield fluorescence images of P-CrkL (Y207) immunostaining and Alexa-568 phalloidin staining in control and knockdown WAVE2 cells. The merged images show P-CrkL (Y207) (green), phalloidin (red) and DAPI stained nuclei (blue). The zoomed images in the bottom row are of P-CrkL (Y207) staining from the regions highlighted by the white boxes in the top row. Scale bar 10 µm. (TIF) [file pone.0064533.s007.tif]

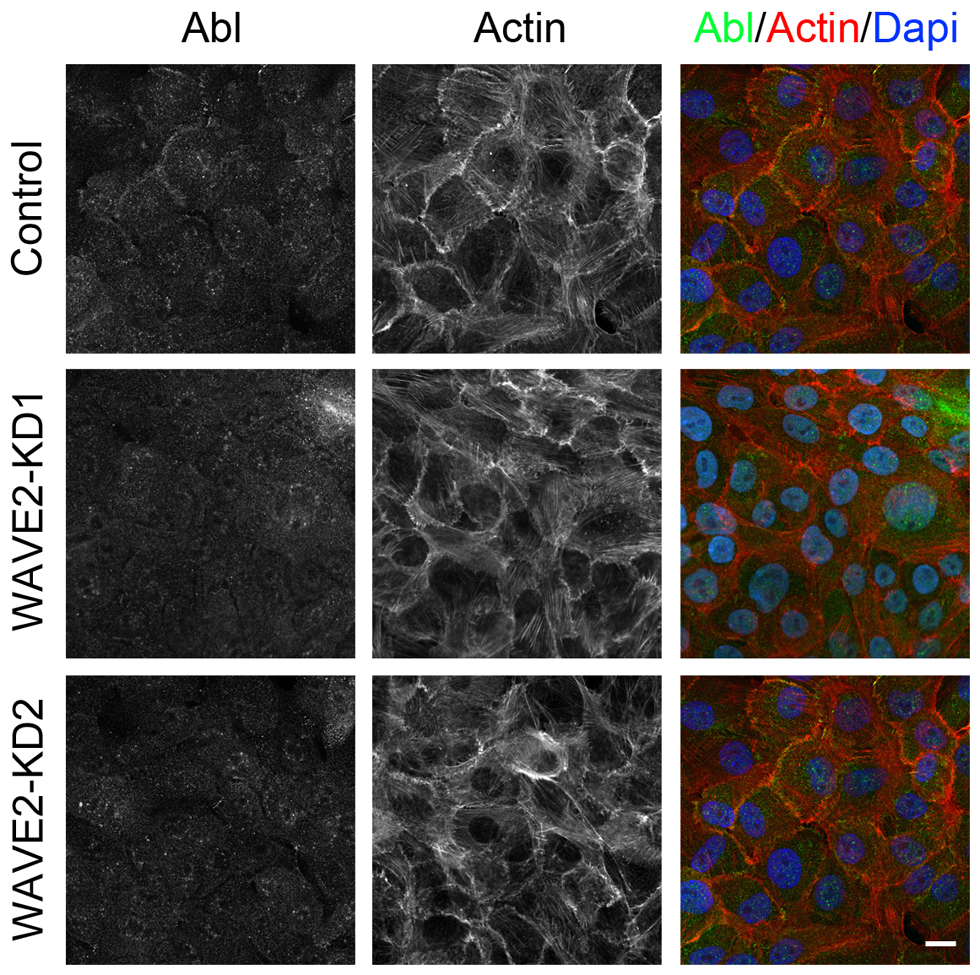

Supplement: Figure S8 — Localization of Abl in control and WAVE2-KD cells in 2D culture. Single confocal images of Abl immunostaining and Alexa-568 phalloidin staining in control and WAVE2-KD cells. The merged image represents Abl (green), phalloidin (red) and DAPI stained nuclei (blue). Arrows point to Abl localization at cell-cell adhesions in control cells whereas arrowheads point to decreased Abl localization at cell-cell adhesions in WAVE2-KD cells. Scale bar 10 µm. (TIF) [file pone.0064533.s008.tif]
